# Antibody-mediated pure red cell aplasia related with epoetin-beta pegol (C.E.R.A.) as an erythropoietic agent: case report of a dialysis patient

**DOI:** 10.1186/s12882-020-01934-2

**Published:** 2020-07-14

**Authors:** Yasuki Shingu, Tomohiro Nakata, Shinji Sawai, Hisaya Tanaka, Osamu Asai, Keiichi Tamagaki, Kimihiko Nakatani

**Affiliations:** 1 Department of Nephrology, Kyoto Yamashiro General Medical Center, 1-27 Kizuekimae, Kizugawa, Kyoto, 619-0214 Japan; 2 Department of Nephrology, Kyoto Prefectural University of Medicine, 465 Kajii-cho, Kamigyo-ku, Kyoto, 602-8566 Japan

**Keywords:** Antibody-mediated PRCA, Epoetin-beta pegol (C.E.R.A.), Cyclosporine, Case report of a dialysis patient

## Abstract

**Background:**

Erythropoietin-stimulating agents (ESAs) are used to treat anemia in patients with chronic kidney disease, enabling maintenance of stable hemoglobin levels and eliminating the need for multiple transfusions. Epoetin-beta pegol (C.E.R.A.) is a continuous erythropoietin receptor activator created by integrating a large methoxy-polyethylene-glycol-polymer chain into the erythropoietin molecule, which provides it with a longer half-life. On rare occasions, cases of antibody-mediated pure red cell aplasia (PRCA) related to ESAs are reported. They are characterized by abrupt onset of severe transfusion-dependent anemia, despite ESA therapy. We herein report a case of antibody-mediated PRCA in a dialysis patient receiving C.E.R.A.

**Case presentation:**

A 44-year-old man with end-stage renal failure had been receiving continuous ambulatory peritoneal dialysis for 2 years. C.E.R.A. was administered subcutaneously as a sole ESA once a month at the hospital since 4 years ago for the treatment of renal anemia and his hemoglobin level was well controlled at 12 g/dl. From 10 months before diagnosis, however, his hemoglobin level suddenly declined, necessitating frequent transfusions. Based on the results of a bone marrow examination and detection of anti-C.E.R.A. antibodies, the patient was diagnosed with antibody-mediated PRCA. After successful elimination of the antibodies using oral prednisolone plus cyclosporine, the patient was re-administrated C.E.R.A. intravenously, as there are few reports of antibody-mediated PRCA related to ESA using that administration route. He responded to the C.E.R.A., and his anemia dramatically improved, eliminating the need for blood transfusions.

**Conclusions:**

This is the first reported case of recovery from an antibody-mediated PRCA with C.E.R.A. after its re-administration following a reversal of the antibody. It has been suggested that the additional large pegylation chain makes C.E.R.A. less likely to trigger antibody generation than other ESAs. Following successful treatment of antibody-mediated PRCA using immunosuppressive therapy, C.E.R.A. can be re-administered intravenously to treat renal anemia.

## Background

Antibody-mediated pure red cell aplasia (PRCA) is a very rare but severe, transfusion-dependent anemia affecting patients receiving treatment with ESA [1]. Antibody-mediated PRCA is characterized by a sudden fall in the hemoglobin concentration, despite ESA therapy, with an almost complete lack of erythroid progenitor cells in an otherwise normal bone marrow. Antibodies to erythropoietin (EPO), detectable in the serum of these patients, neutralize not only the biological activity of therapeutic ESA, but also endogenous EPO [2, 3].

The incidence of PRCA related to ESA therapy is reported to be 0.02–0.03 per 10,000 patient-years [4]. Especially, the peak incidence of PRCA related to ESA therapy occurred during 2002–2003 [1]. The majority of those cases were caused by a preparation of epoetin-alfa (Eprex/Erypo) [5, 6], though a small case series caused by epoetin-beta was also reported [7–10]. This transient increase of PRCA related to Eprex during 2002–2003 was associated with the use of the polysorbate-80(PS-80) formulation in prefilled syringes with uncoated rubber stoppers [6, 11]. It is proposed that the compounds with adjuvant activity leached by PS-80 from plastics and rubber materials in uncoated stoppers induce an anti-EPO immune response [11, 12]. Alternatively, it is proposed that the use of PS-80 and glycine instead of human albumin make the epoetin α formulation less stable if exposed to high temperatures. This may result in the formation of aggregates of EPO following subcutaneous injection, thus increasing the risk of anti-EPO antibody development [13]. On the other hand, Epoetin-beta pegol (continuous erythropoietin receptor activator; C.E.R.A.) is a third-generation ESA developed by attaching a large pegylation chain to epoetin-beta, and is more stable if exposed to high temperatures than other ESAs [14, 15]. It is thought that the additional pegylation chain may make this molecule more resistant to antibody generation than the other ESAs, because protein pegyltion tend to decrease immunogenicity, presumably as a result of steric hindrance that prevents the immune system from recognizing the therapeutic protein as foreign [16]. Actually, as of July 2019, there have been only 10 reports of confirmed antibody-mediated PRCA related with C.E.R.A. according to regulatory documents of Chugai Pharmaceutical Co. Ltd.. Moreover, a search of the literature using the PubMed system turned up no reported cases of antibody-mediated PRCA related with C.E.R.A. This is the first in the literature that reported a case of antibody-mediated PRCA associated with administration of C.E.R.A. in a continuously ambulatory peritoneally dialyzed patient.

## Case presentation

**Fig. 1:**
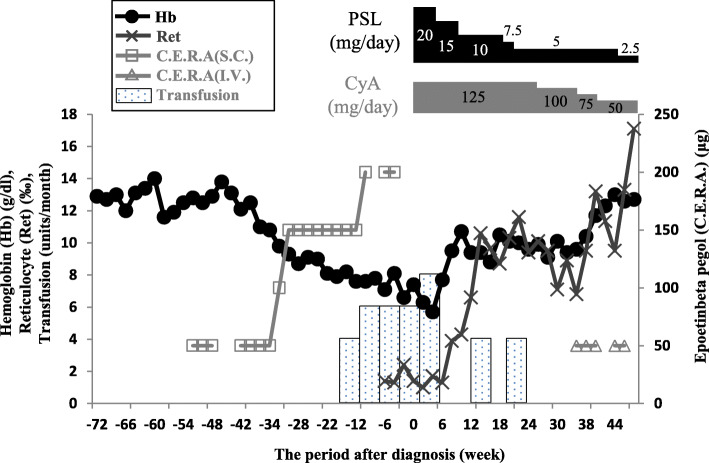
Clinical course of the patient: black circle line, changes in serum hemoglobin (Hb); black cross line, changes in reticulocyte counts (Ret); gray square line, subcutaneous (S.C.) injection of C.E.R.A.; gray triangle line, intravenous (I.V.) injection of C.E.R.A. Inset bar graph: red blood cell transfusions (Transfusion). C.E.R.A.: Epoetin-beta pegol, PSL: Prednisolone, CyA: cyclosporine

A 44-year-old man with chronic renal failure was admitted to our hospital because of the marked progression of anemia. He had been on continuous ambulatory peritoneal dialysis since 2 years ago for the treatment of end-stage renal failure due to chronic pyelonephritis. He had also been subcutaneously administered C.E.R.A. as a sole ESA once a month at the hospital since 4 years ago for the treatment of renal anemia. At the hospital, C.E.R.A. had been kept refrigerated until it was administered. He was also being subcutaneously administered C.E.R.A. at a dose of 50 μg per month, which controlled his hemoglobin level at 12 g/dl. From 10 months before diagnosis, however, his hemoglobin level suddenly began to decline, reaching as low as 6 g/dl. Increasing the C.E.R.A. dose did not slow the progression of anemia, and frequent transfusions were required (Fig. 1). On admission, his body temperature was 36.8 °C and blood pressure was 110/60 mmHg. His palpebral conjunctiva was serious anemic and bilateral lower leg edema was not observed. He did not present with a symptom of throat infection, and did not have a past infection before admission. Laboratory results were as follows: red blood cell count, 2.29 × 10^6^ /μl; hemoglobin, 7.1 g/dl; hematocrit, 20.7%; mean corpuscular volume, 90.4 fl; reticulocyte count, 3.2 × 10^9^ /l; white blood cell count, 4830 /μl with normal differentials; platelet count, 1.2 × 10^5^ /μl; lactate dehydrogenase, 197 IU/l; total bilirubin, 0.34 mg/dl, which showed a normocytic anemia accompanied with reticulocytopenia and no evidence of active hemolysis. Anti-nuclear antibodies and anti-DNA antibodies were negative. The patient was also negative for IgM antibodies to parvovirus B19. Computed tomography of the chest did not reveal thymoma. Further examination, including of the upper gastrointerstinal tract and a fecal occult blood test, could not detect an apparent bleeding source. The dialysis was adequate and he did not have a significant mineral bone disorder. The ferritin level was more than 500 ng/l, with transferrin saturation at 91.8% thanks to the frequent transfusions, and the folate level was normal. At the peak of the condition, the patient needed packed red blood cell transfusions almost every 2 weeks (Fig. 1).

Bone marrow examination confirmed severe erythroid hypoplasia with entirely normal white cell and platelet precursors. Antibody-mediated PRCA was therefore suspected. The level of anti-C.E.R.A antibodies was assessed by Chugai Pharmaceutical Co. Ltd. Kamakura Research Laboratories using an enzyme-linked immunosorbent assay, which used the patient’s own serum as the primary antibody [17]. The results showed positivity for both anti-epoetin-beta and anti-C.E.R.A antibodies (anti-epoetin-beta: 1.1205 titer (normal range < 0.3973), anti-C.E.R.A.: 0.0275 titer (normal range < 0.0123)) (Table 1). Based on those findings, the patient was diagnosed with antibody-mediated PRCA associated with C.E.R.A.

**Table 1:** The titers of anti-C.E.R.A. and anti-epoetin-beta antibodies

	Anti-C.E.R.A. antibody				Anti-epoetin-beta antibody			
	1^st^ assay		2^st^ assay		1^st^ assay		2^st^ assay	
	Titer	Normal range	Value (%)	Normal range	Titer	Normal range	Value (%)	Normal range
At diagnosis	0.0275	≦ 0.0123	79.6	≦ 55.6	1.1202	≦ 0.3973	94.4	≦ 15.6
On the 20th week after the therapy	0.0075	≦ 0.0125			0.125	≦ 0.1858		
On the 32th week after the therapy	0.015	≦ 0.0235			0.1045	≦ 0.196		
On the 20th week after the re-administration	0.0165	≦ 0.0283			0.104	≦ 0.1633		

1^st^ assay represents the titer of anti-C.E.R.A. or anti-epoetin-beta antibody determined using an enzyme-linked immunosorbent assay

2^st^ assay shows the decline rate in the titer of anti-C.E.R.A. or anti-epoetin-beta antibody when excess recombinant C.E.R.A. or epoetin-beta proteins was added

The patient’s clinical course is depicted in Fig. 1. Administration of ESA was stopped. The option of renal transplantation was explored, but unfortunately no suitable kidney donor was found. Therefore, oral administration of prednisolone and cyclosporine was commenced, resulting in a gradual increase in his reticulocyte count and a decreased in the frequency of the red blood transfusions. By the 20th week of therapy, both anti-epoetin-beta and anti-C.E.R.A. antibodies were undetectable, which was also confirmed on the 32nd week (Table 1). At that time, we commenced intravenous C.E.R.A. 50 μg monthly. He tolerated the C.E.R.A. injection well, with no allergic reaction. The patient’s hemoglobin level increased, enabling red blood transfusions to be stopped. Since then, his hemoglobin level has been stable at 12 mg/dl (Fig. 1), and 20 weeks after re-introduction of C.E.R.A., the patient remains negative for both anti-epoetin-beta and anti-C.E.R.A. antibodies (Table 1).

## Discussion and conclusions

We present here the first case of antibody-mediated PRCA caused by antibodies to both epoetin-beta and C.E.R.A after administering C.E.R.A subcutaneously, in which these antibodies titers were evaluated over time and re-administration of C.E.R.A. could successfully treat PRCA after antibodies reversal. PRCA is a primary hematologic disease, but the acquired form of PRCA has been reported to be caused by parvovirus infection, leukemia, lymphoma, thymoma, autoimmune diseases, severe malnutrition, or exposure to certain drugs and toxins [18]. After exclusion of other causes of ESA hyporesponsiveness, ESA-induced PRCA is diagnosed based on the presence of reticulocytopenia (< 1 × 10^10^/l) and neutralizing anti-EPO antibodies [18]. Circulating anti-EPO antibodies are typically detected using an immunoassay (radioimmunoprecipitation or enzyme-linked immunosorbent assay) or surface plasmon resonance methods [18]. In the present case, a diagnosis of anti-C.E.R.A. antibody-mediated PRCA was made based on bone marrow examination revealing reticulocytopenia as well as detection of both anti-epoeti-beta and anti-C.E.R.A. antibodies in the serum and exclusion of other causes of PRCA.

Antibody-mediated PRCA is very rarely self-limiting and usually necessitates therapeutic intervention. The most important initial treatment is to stop administration of ESA therapy which causes immunogenicity, and to treat the anemia with red blood cell transfusion. The most effective treatment is kidney transplantation because antibodies to EPO can be reversed post-trasplantation [19, 20]. A retrospective study previously showed patients of antibody-mediated PRCA who received a kidney transplant were cured within 1 month and achieved normal hemoglobin concentrations. When accompanied by adequate antirejection therapy, kidney transplantation can treat antibody-mediated PRCA in patients with chronic renal failure by suppressing antibody production and recovering EPO secretion from the transplanted kidney [19–21]. On the other hand, in cases of antibody-mediated PRCA, antibodies or cytotoxic T lymphocytes attack erythroid precursors [1]. It is therefore treated with immunosuppressive agents, including corticosteroids, cyclosporine, cyclophosphamide, rituximab, and intravenous immunoglobulin [20, 22–24]. The reported response rates to cyclosporine and prednisolone (0.5–1.0 mg/kg) in patients with antibody-mediated PRCA related to ESA are 67 and 56%, respectively [25]. In our case, administration of oral prednisolone (30 mg/day) and cyclosporine (100 mg/day) was effective, and anti-C.E.R.A. antibodies were undetectable after 20 weeks of treatment. Thereafter, the patient was less dependent on blood transfusions. In general, intravenous administration of a protein is much less likely to evoke an immune response than intranasal, intramuscular or subcutaneous administration [26]. We therefore re-administered C.E.R.A. intravenously, after which we did not detect the reappearance of anti-C.E.R.A. antibodies, even after 20 weeks of re-administration, and the patient’s hemoglobin levels were well controlled at 12 mg/dl.

This case demonstrates the potential multifactorial nature of anemia in dialysis patients receiving an ESA and highlights the importance of investigating antibody-mediated PRCA related to ESA, including of C.E.R.A. In addition, we recommend re-administration of ESA by intravenous injection when anti-EPO antibodies are below or around the lower limit of detection.

## Data Availability

The datasets supporting the conclusions of this article are included within the article.

## Abbreviations

ESA: Erythropoietin-stimulating agent
C.E.R.A.: Epoetin-beta pegol (continuous erythropoietin receptor activator)
PRCA: Pure red cell aplasia
